# “It Should Be a Priority”: Lessons Learned by Head Start Leaders, Staff, and Parent Facilitators Delivering a Multi-Site Parent-Centered Child Obesity Prevention Intervention

**DOI:** 10.3390/nu17061063

**Published:** 2025-03-18

**Authors:** Cristina M. Gago, Alyssa Aftosmes-Tobio, Natalie Grafft, Kirsten K. Davison

**Affiliations:** 1 Department of Community Health Sciences, Boston University School of Public Health, Boston, MA 02119, USA; 2 School of Social Work, Boston College, Chestnut Hill, Boston, MA 02467, USA; alyssa.aftosmes@gmail.com (A.A.-T.); grafftn@bc.edu (N.G.); davisonk@bc.edu (K.K.D.)

**Keywords:** early childcare, education, nutrition intervention, implementation science, parenting, community-based participatory research

## Abstract

**Background**: Head Start, a federally funded preschool for low-income families, offers a unique space for interventionists to equitably reach parents and children, and promote healthful behavior for chronic disease prevention. However, determinants of implementation in this context remain understudied, hindering opportunities for improvement. We aim to identify organization-level factors affecting implementation of an obesity prevention program, as relayed by implementation partners at Head Start. **Methods**: Communities for Healthy Living (CHL), designed and implemented with Greater Boston Head Start (*n* = 16 programs across *n* = 2 agencies), is a cluster-randomized obesity prevention trial offering enhanced nutrition support, media campaign, and a parenting program. The current study draws on two years (2017-19) of data collected from Head Start implementation partners. Pre-implementation, staff completed anonymous surveys: implementation readiness (*n* = 119), staff training evaluation (*n* = 166), and facilitator training evaluation (*n* = 22); response frequencies were tabulated. Mid-implementation, staff and leadership participated in focus groups (*n* = 3 groups with *n* = 16 participants) and interviews (*n* = 9); transcripts were analyzed using a deductive-inductive hybrid approach, grounded in the Consolidated Framework for Implementation Research. **Results**: Most staff strongly agreed or agreed they understood their role (98.8%), planned on recruiting parents (98.2%), and reported commitment to implementation (92.5%); however, fewer identified CHL as a priority (69.7%) and were confident in their ability to coordinate efforts (84.9%), handle challenges (77.3%), and receive support (83.2%). Thematic analysis yielded implementation facilitators, including mission alignment, partner engagement in design, allocation of intervention-specific resources, and expressed leadership support. Barriers included strains imposed on staff workflow, a lack of shared responsibility, and challenges in coordinating CHL activities amidst competing Head Start programs. **Conclusions**: Responsive efforts to address deliverer-identified barriers to implementation may include reducing intervention impact on preexisting workflow, as well as clearly distinguishing intervention activities from preexisting Head Start programs.

## 1. Introduction

Chronic disease is the leading cause of morbidity and mortality in the United States (U.S.) [1]. Disparities in chronic disease risk—by socioeconomic status [2], race and ethnicity [3]—emerge early [3,4] and persist across the lifespan [5,6] as a result of inequitable structural barriers to social, economic, and environmental opportunity [7,8], alongside heightened stress levels and reduced access to healthcare [9]. Interventions targeting modifiable behavioral risk factors early in life [10] are urgently necessary [11,12,13]—especially those which authentically engage and serve health disparity populations most affected by chronic disease burden [3].

One promising setting for such behavior change intervention efforts—including obesity prevention interventions—is that of early care and education (ECE) [14,15,16,17]. Not only do ECEs reach over 60% of children age 3–5 years in the U.S. [18], but they also offer a longitudinal connection with parents and the larger family system. Consequently, an abundance of ECE-based health promotion and behavior change interventions have emerged in the past fifteen years [14,19].

Although parent-engaged approaches to child health behavior change are considered the “gold standard” [20], few ECE studies have directly, effectively, and sustainably engaged parents as agents of change in child health behavior. This is attributed to notable challenges engaging parents of young children, including but not limited to a lack of parent and ECE staff time, competing work and caregiving responsibilities, and a lack of childcare outside school hours [21,22]. Instead, most ECE-based interventions have focused on teacher and child behavior intervention [23]. Fewer still have used a community-engaged participatory (CBPR) [24] approach to for intervention design and delivery [25,26,27].

In response, our academic research team partnered with parents, staff, and the leadership of Greater Boston Head Start—a federally funded school readiness program for young children (aged three to five years) of low-income households disproportionately affected by chronic disease [28]. Together, we adapted and implemented Communities for Healthy Living (CHL), a multi-component intervention to support parental empowerment and healthy parenting behaviors among low-income families across 16 programs (adaptation: 2016–2017, implementation: 2017–2020) [29], involving enhanced nutrition support, a media campaign, and a 10-week, opt-in parenting program.

Previously published findings suggested that parents who enrolled in CHL’s parenting program (vs. not) demonstrated significant increases in parental empowerment [30,31]; further, both parenting program participants [32] and facilitators [33] reported high program acceptability and appropriateness. While those exposed to lower-intensity CHL intervention components (e.g., enhanced nutrition support and media campaign) reported higher odds of children meeting three health behavior recommendations (i.e., sugar-sweetened beverage intake, water intake, and screen time), intervention exposure was not associated with clinically significant changes in child weight status.

Mixed results like these are common [34,35], despite mounting evidence supporting the promise of ECE-based interventions for early childhood health promotion intervention [36]. Further, those studies which do observe evidence of effectiveness often find that changes are not sustained long-term [37]. Contextual implementation determinants which are potentially driving these inconsistent and poorly maintained results remain understudied—particularly among CBPR interventions targeting parent behavior in low-resource ECE contexts [23,38]. Specifically, as noted in a recent scoping review of determinants underlying implementation of ECE-based interventions, there are limited data from evaluations grounded in theory, models, or frameworks, and from interventions designed to serve low-income or minorized populations specifically [23]. These research gaps not only prevent the advancement of intervention revision efforts, but they also impede progress in closing striking disparities in chronic disease risk and outcomes [3].

The current multimethod study aimed to fill this knowledge gap by summarizing contextual determinants (i.e., factors driving the success or failure of implementing an intervention) experienced and reported by Head Start leaders, staff, and parent facilitators over two years of intervention implementation (2017–2019).

## 2. Materials and Methods

### 2.1. Study Design

The current study describes findings from pre-implementation surveys and mid-implementation-interviews with Head Start staff, leadership, and facilitators (parents and staff) involved in the CHL intervention implementation (2017–2019). The CHL intervention is a program-level cluster-randomized pediatric obesity prevention intervention designed in partnership with Head Start (2016–2019) to support parental empowerment and healthy parenting behaviors among low-income families. Details on CHL protocol [29,39] (e.g., theoretical framing, participatory methods, intervention components) and impact evaluation findings [30] have been previously reported.

### 2.2. Intervention Development

Aligned with a CBPR [24] approach, CHL intervention design, implementation, and evaluation were driven by community-researcher co-leadership. For the first year, Head Start convened two Community Advisory Boards (CABs) of parents and staff, who met regularly to adapt study materials from a 2009 upstate New York pilot study [40] to fit the Greater Boston context. Key changes included extending the parenting program (PConnect) to 20 h over 10 weeks and adding nutrition support for Head Start staff. Media resources were expanded from posters to include brochures, social media, and online platforms. Ultimately, the adapted intervention involved three components: enhanced nutrition support, a media campaign, and a 10-week opt-in parenting program (PConnect), which have been described previously [29]. The revised program and evaluation surveys were then pilot tested in spring 2017 before the randomized trial in fall 2017.

Prior to intervention implementation, the study manager, with oversight from the study statistician, randomly assigned Head Start programs to one of three intervention start times. Over the next three years, the adapted intervention was implemented across 16 programs, via stepped wedge design [41], with the support of Head Start staff and leadership. Aligning with CBPR principles, financial resources were shared through subcontracts; an intervention coordinator was hired by Head Start partners to support translating research plans into implementation efforts. Primary study outcomes included child weight status and body mass index z-score. Secondary outcomes included child health behaviors (i.e., dietary intake, physical activity, screen use, and sleep), parenting practices across the same domains, and parent empowerment.

### 2.3. Theoretical Framework and Intervention Components

**Figure 1:**
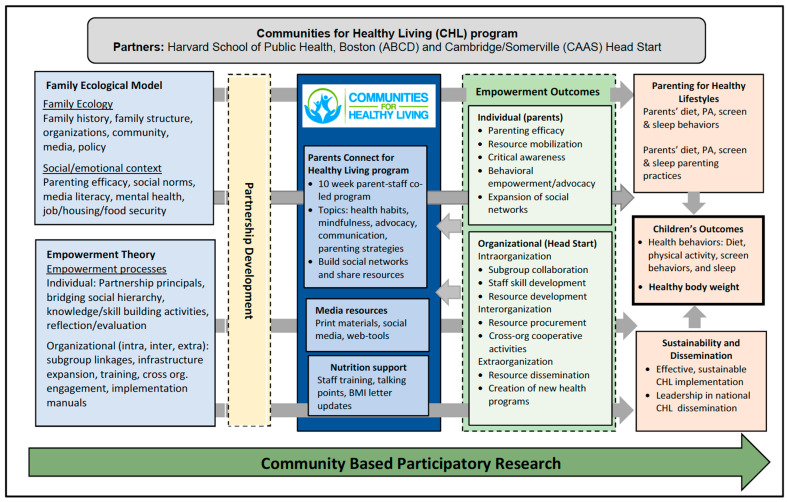
The Communities for Healthy Living intervention theory of change.

Grounded in empowerment theories [42] and the Family Ecological Model [43], the CHL intervention’s theory of change [29] (Figure 1) posited that increases in parental empowerment (i.e., the process by which parents realize control and take action for child health) [42,44,45,46] and healthy weight-related parenting practices (i.e., behaviors to shape child health) [47] would be associated with positive changes in child health behaviors and weight status [46]. Intervention components described above directly align with this theory of change.

Briefly, the enhanced nutrition support involved implementing standardized procedures for height and weight measurements of children and counseling for parents, developing a technical manual for anthropometric measurement and questionnaire administration, creating visual aids to facilitate questionnaire administration, delivering ongoing staff training, and revising child health screening reports. The media campaign involved redesigning brochures and flyers, sharing community-specific information and resources through online platforms, and creating a novel online neighborhood resource map. Finally, a 10-week parenting program was designed to cover content areas critical to child health (e.g., parenting practices, parent and child health behaviors, mindfulness, advocacy, and communication), as well as support skill development in social networking and resource sharing.

### 2.4. Data Collection

**Figure 2:**
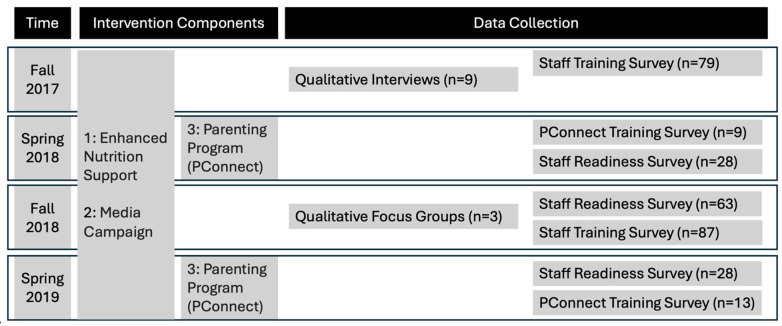
Overview of Community for Healthy Living intervention components and data collection timeline.

Data collection procedures were previously described in detail [39] and the data collection timeline is summarized in Figure 2. Briefly, data were collected through surveys, interviews, and focus groups between September 2017 and June 2019, in alignment with fall (September–December) and spring (April–June) semesters at Head Start.

Specifically, three anonymous surveys were administered to eligible Head Start staff and PConnect facilitators; these surveys included the Staff Training Survey, the PConnect Training Survey, and the Staff Readiness Survey. The Staff Training Survey (*n* = 5 items) was administered via paper to Head Start staff in fall 2018 and 2019 at the end of CHL intervention training offered before intervention initiation; survey items were developed to measure clarity and complexity of staff role in the CHL intervention and self-efficacy using CHL intervention resources. Second, the PConnect Training Survey (*n* = 32 items) was administered in Spring 2018 and 2019 via paper at the end of the three-day training for PConnect facilitators (Head Start parents and staff) covering PConnect program content and facilitation skills; items were developed to measure training effectiveness, training quality, and the quality of PConnect program materials. Third, via convenience sampling, all Head Start staff at participating intervention sites were invited via email broadcast and in-person staff meetings to complete the Staff Readiness Survey (*n* = 29 items adapted from the validated Organizational Readiness for Implementing Change (ORIC) measure [48]) via paper or Research Electronic Data Capture (REDCap); this survey was administered biannually in 2018 and 2019 and survey items were developed to measure staff readiness to implement the intervention, staff use of CHL materials, as well as perceived quality and usefulness of CHL materials.

Via purposive sampling, key Head Start staff and leadership involved in the implementation of CHL were selected and invited to participate in qualitative interviews (*n* = 9 staff) in fall 2019 and focus groups (*n* = 2 with *n* = 2 leaders and *n* = 14 staff) in fall 2018. Participants included Head Start program directors, supervisors and family engagement managers, Head Start nutritionists, Head Start health service team members, and CHL coordinators. Interviews and focus groups lasted between 30 min and one hour. Interviews were conducted via phone, and focus groups were conducted in person at Head Start administrative offices. All interviews and focus groups were audio recorded, and transcribed by an offsite company (Landmark Associates, Inc., Phoenix, AZ, USA).

### 2.5. Ethics

All surveys, interviews, and focus groups were conducted by trained research staff. Before survey completion, all participants reviewed information about the study and marked a checkbox as indication of consent. Before interview or focus group completion, participants received a key information sheet, reviewed information about the study verbally, and provided verbal consent. Study protocols were approved by the ethics review boards at Harvard University and Boston College. The trial protocol was registered at ClinicalTrials.gov (NCT03334669).

### 2.6. Conceptual Framework

Given the lag in translating behavior change research into practice in the context of ECEs [23], we grounded our evaluation in the Consolidated Framework for Implementation Research 2.0 (CFIR). CFIR represents one of the most widely published implementation science determinant frameworks, programing attention around innovation recipients (e.g., parents) and determinants of equitable implementation [49]. Specifically, we focused on three CFIR domains (e.g., levels), including that of the individual (e.g., Head Start staff and leadership), the innovation (e.g., the CHL intervention), and the inner setting (e.g., Head Start sites).

### 2.7. Analysis

We applied a convergent, mixed-methods approach, wherein quantitative and qualitative data were collected and analyzed independently and subsequently integrated via joint display [50]. (CG and Survey responses were downloaded from REDCap; frequency (*n*, %) and mean scores were tabulated in R 4.2.2 (R Core Team, 2022). Simultaneously, qualitative data were analyzed by two trained qualitative researchers AAT) via thematic analysis. Specifically, the primary coder (CG) used a deductive approach to capture implementation determinants through a preliminary coding structure informed by the CFIR. Both coders then reviewed the initial codebook and used inductive (open and axial coding) methods to independently code the same two transcripts. The coders then met over video conference to review and revise the codebook, as well as reconcile coding differences through comparison and discussion. After applying the revised codebook to independently code another two transcripts and revise the first two, they then met again to compare and reconcile differences; at this point, coding was deemed consistent (though comparison and discussion) and no further changes to the codebook were required. The revised codebook was then used to independently code the remaining transcripts, and a final set of themes were collaboratively generated through excerpt review and discussion over video conference. A total of *n* = 12 transcripts were coded for analysis, including *n* = 9 staff interviews and *n* = 3 focus groups (with a total of *n* = 16 participants; range: 2–10 participants). All analyses were conducted in Dedoose (version 9.0.17, Los Angeles, CA, USA).

## 3. Results

### 3.1. Sample Characteristics

Briefly, most survey respondents identified as female (61.3% of staff and 100% of PConnect facilitators, Table 1). Approximately one in three staff respondents and one in four PConnect facilitators identified as Hispanic (33.6%), one in five (19.3%) as non-Hispanic (NH) Black, and one in five as NH Asian; among PConnect facilitators, fewer identified as Hispanic (27.3%) and more as NH Black (31.8%). Similar frequencies of staff and facilitators had earned an undergraduate or graduate degree (49.6% of staff and 40.9% of facilitators). A majority of staff had been at Head Start for 10 years or more (50.5%) while most facilitators had been there less than 4 (72.6%).

#### 3.1.1. Theme 1 (CFIR’s Mission Alignment): CHL Intervention Activities Align with the Head Start Mission

*“With [Head Start’s] mission [to support] families, I think [CHL] gives you one more way to connect with families.”*—Head Start leadership

Head Start staff and leadership described nutritional health not only as an important community need, but as a prerequisite for Head Start’s mission of promoting school readiness (e.g., “If you don’t take care of the basic needs of children and families… [it] doesn’t matter what you teach them—it’s not gonna sustain.”). This same synergy was also reflected in survey responses, with 93.2% of staff agreeing that the CHL intervention addresses an important need for the community and 94.9% agreeing that the CHL intervention will be effective in improving family and child health (Table 2 and Table S1). Beyond school readiness and health outcomes alone, Head Start staff viewed CHL’s activities as an additional platform for connecting with parents, which is central to Head Start’s mission to engage family systems in child development (“It’s everybody’s business when it comes to parent engagement and recruitment, helping families”). This agreement was also reflected in survey responses, through which 95.5% of PConnect facilitators agreed that PConnect would help them connect with parents.

#### 3.1.2. Theme 2 (CFIR’s Engaging): Participation in the Design Process Results in High-Quality Materials

*“I feel like the resource map was a really great… tool that I could share with those specific staff members. It helped [staff] support their families.”*—Head Start Staff

Interview responses suggest that engaging with the Head Start leadership, staff, and parents was critical for improving the quality, appropriateness, and acceptability of materials. Head Start staff consistently reported that participatory methods resulted in the development of better materials, including staff talking points, brochures, and online neighborhood resource maps (e.g., “Any changes that we suggested were done. In the meeting we all talked about it. Then the next meeting, we will see what they work on and results.”). Further, Head Start staff appreciated the receptivity of the research team to changes (e.g., “You guys have been very receptive in listening to everything”). This appreciation was also reflected in the survey responses, with 66.7% of Head Start staff strongly agreeing they would use the Healthy Habits brochures as a resource. However, mid-implementation, reports of material utilization were more varied, with 33.3% reporting they consistently use the Staff Talking Points, 66.7% Healthy Habits brochures, and 50.7% using the Neighborhood Resource Map (Table S2). Despite this variable uptake, most Head Start staff rated the staff talking points, healthy habits brochures, and posters as useful.

#### 3.1.3. Theme 3 (CFIR’s Available Resources): Allocation of CHL-Specific Resources Is Necessary for Success

*“We are running by a shoestring budget… you definitely need some funding.”*—Head Start Staff

Head Start staff and leadership reflected on the importance of allocating intervention-specific resources and staff to alleviate Head Start employee burden. Further, they described the challenges associated with long-term intervention planning in the context of short-term funding cycles (e.g., “We’ve been having that support from the [university] people and then once they’re no longer involved… I’m thinking money is the future of the grant. I’m thinking about it a lot.”). Specifically, leadership applauded the positive impact that hiring a CHL-specific coordinator posed on staff implementation burden (e.g., “[The CHL intervention coordinator] is extremely helpful. She’s there. She’s around. She kind of takes the burden off people”).

#### 3.1.4. Theme 4 (CFIR’s Relative Priority): Demonstrated Support from Leadership Is Necessary for Staff Buy-In and Prioritization

*“If you don’t have the support from the director, it doesn’t work.”*—Head Start Staff

Many Head Start staff and leadership noted the need for greater program visibility and involvement among management. Achieving this would not only ensure that staff felt support for their implementation efforts, but it would also help to facilitate greater parent engagement with the program (e.g., “If the program director has an understanding of what’s happening… that could be included in the calendar, if she was better aware of what was going on with CHL”). This lack of CHL intervention visibility was also reflected in survey responses, with only 28.6% of staff strongly agreeing that they were confident they would be supported in CHL implementation and 19.3% strongly agreeing CHL is a priority at their program. Beyond prioritization alone, Head Start staff viewed leadership and management involvement as critical for building and maintaining accountability structures; for example, staff reported that lack of authority over others limited staff ability to coordinate CHL intervention activities with other staff, calling for management and leadership to step in (e.g., “I’m not an authority—I’m not anyone’s boss, so it’s hard to make—have any authority to tell people they need to be doing things”). Lack of alignment between CHL intervention activities and staff responsibilities further hindered CHL intervention activity coordination (e.g., “My supervisor might say that it has, potentially, taken away from other things… because [CHL] wasn’t technically my job description.”).

#### 3.1.5. Theme 5 (CFIR’s Deliverer Opportunity): CHL Strains Workflow but Offers Benefits

*“I feel like [the CHL intervention] needs to be something that is added in to our responsibilities, I guess.”*—Head Start Staff

Head Start staff perceived CHL intervention tasks as additional burdens beyond that required of their already high responsibilities and expectations; this directly contrasted the intervention designers’ intention of integration. In response, staff called for the need to formally align CHL intervention activities with staff responsibilities or formally add CHL intervention activities to job descriptions and expectations. This same sentiment was also reflected in survey responses, with one-in-four surveyed staff (22.7%) strongly agreeing that the CHL intervention fits within their current job responsibilities and 32.8% strongly agreeing that they want to implement the CHL intervention. Still, despite the time commitment it involved, staff did report benefits of supporting the CHL intervention implementation; for example, 77.3% of PConnect facilitators surveyed agreed the experience of facilitating would help them professionally.

#### 3.1.6. Theme 6 (CFIR’s Teaming): Lack of Shared Responsibility Amidst Frequent Turnover Hindered CHL Implementation

*“We’ve lost a few staff over the last couple of years… if you don’t have someone who has seen it through who can be a mentor to other people, it can be tough.”*—Head Start Staff

Overall, Head Start staff described the lack of shared responsibility as a barrier to implementation. This was attributed to two key factors: lack of staff engagement and turnover, especially early in the intervention, among leadership and champions involved in intervention development. Regarding staff engagement, staff and leadership described lack of cohesion as a barrier to CHL intervention implementation (e.g., “I think it needs to be more cohesive with staff being included”); in response, staff and leadership described the need for authentically and actively engaging as many staff as possible in design and implementation (e.g., “Incorporating staff, I think, needs to be on the front burner”). Regarding turnover, staff described inconsistent training for off-cycle hires and a lack of long-term involvement with the CHL intervention as barriers to implementation. For example, pervasive lack of training and experience with the CHL intervention resulted in implementation burden falling almost entirely on a few key staff members (e.g., “[When a team member does not] necessarily feel empowered to do [an activity] … I think it falls on my shoulders, or it doesn’t happen”). This lack of teaming was also reflected in survey responses. While nearly all staff (92.5%) reported they were committed to implementing CHL, the degree of commitment varied, with only 30.3% strongly agreeing versus 62.2% agreeing they were committed. A few more staff strongly agreed they wanted to implement CHL (32.8% versus 59.7% agreed), indicating a potential disconnect between desire to support and capacity to do so.

#### 3.1.7. Theme 7 (CFIR’s Compatibility): Challenges Coordinating Competing Programs

*“We have classes, consults, parent meetings, different workshops so the parents want to do everything, but they can’t… that could be affecting attendance.”*—Head Start Staff

Head Start staff reported that scheduling CHL was often a challenge amidst the rich array of other programs offered at Head Start—not only in terms of allocating staffing to cover simultaneous programming, but also finding space and directing parents to the appropriate programs (e.g., “I feel like…trying to schedule [CHL] around other programs… that’s the only real problem with the CHL grant.”). Even in cases where parents were interested in multiple programs, staff noted they would have to choose one, ultimately hindering attendance across all competing programs, not just the CHL intervention. Further, the wide array of frequently changing options made it hard for staff to keep up to date, leaving both staff and parents overwhelmed with information (e.g., “They would be confused because we have so many meetings.”). Without distinguishing CHL as a unique and important program with specific goals, staff were unable to distinguish and elevate it in the context of other ongoing programs. This was reflected in the survey data as well, with 21.0% strongly agreeing they could help coordinate efforts.

## 4. Discussion

While most prior studies targeting obesity prevention in early childhood have focused on child-programed approaches [51], the CHL intervention partnered with ECE leadership, staff, and parents to address larger family ecologies through parent behavior change [43]. This study is among the first to explore implementation partner perspectives on contextual determinants to parent-programed obesity prevention intervention implementation in the context of Head Start, despite burgeoning evidence supporting the promise of intervention research in this context [23]. Aligning with recent literature on contextual determinants of ECE intervention sustainability [51], findings synthesized through our multimethod approach suggest facilitators to intervention implementation include mission alignment, authentic engagement, resource co-investment [52], and leadership buy-in as facilitators to intervention implementation. On the other hand, partners also noted several key barriers to implementation, including misaligned expectations, siloed responsibility, and challenges coordinating competing programs [53].

From these findings and prior literature, several key practice recommendations were generated. First, mission and expectations between the intervention and partner organizations must be aligned. Further, these must be communicated clearly, consistently, and repeatedly over the intervention life course for partner staff and leadership to justify intervention prioritization and distinction amidst competing programs offering clearer alignment. For example, materials disseminated in the context of Head Start must directly and clearly support school readiness [54]. This may also support program visibility and buy-in amongst leadership [55], which could facilitate management of staff expectations and shared responsibilities [56] related to intervention activities.

Second, following a CBPR approach [57], all stages of intervention design and implementation should be iteratively revised [58] in partnership with engaged Head Start leadership and staff. Not only does iterative engagement process foster partner buy-in, but it also ensures that materials fit the unique needs of the community, thereby optimizing intervention implementation and, ultimately, effectiveness [24].

Third, in line with CBPR principles, intervention designers should invest in and share resources for long-term intervention sustainment [59,60]. In particular, Head Start staff and leadership applauded investment in personnel for intervention coordination. However, anticipation of coordinator loss at grant’s end was considered detrimental for long-term intervention sustainment [61]. Intervention efforts in ECEs require efficient methods for assessing, establishing, and maintaining clear priorities to ensure appropriate allocation of limited funding, strict scrutiny of external resources, and clarity about staff roles, responsibilities, and the interconnections across program components. In cases where long-term intervention-specific staffing is untenable, train-the-trainer models [62] may be appropriate.

Beyond hiring additional staff, interventionists must also intentionally work with ECE leadership to align intervention engagement opportunities [63] with current staff expectations and workflows. Achieving this will not only ensure that staff could get more involved, but also that they are able to access the concrete support necessary for success. To further support responsibility sharing and prevent tasks from falling on a select few, interventionists must simultaneously work with ECE leadership to build supervisory structures for intervention tasks in support of implementation oversight and authority [64]. This is particularly important in the context of ECE, where money is stretched thin and turnover rates are high [65,66]. Beyond supporting intervention sustainability alone, these findings suggest that public investment in professional development opportunities, behavioral health initiatives, and mentorship services is critical for supporting staff retention and maintaining a stable ECE workforce [67].

Further, applying a policy, systems, and environment approach—which reshapes the contexts in which children grow—may provide a sustainable model for promoting healthful behavior change while reducing ECE staff burden [13,68,69,70,71]. Examples of such interventions include connecting families with existing social and economic resources (e.g., nutrition assistance programs, legal services, job training programs), increasing healthy food options at ECEs and across neighboring retailers, providing transportation and childcare supports for parents to attend programs like PConnect, and build strategic partnerships with universities to support participatory grant writing and program development [70].

Several key limitations should be noted. First, data were collected before the Coronavirus disease 2019 (COVID-19) pandemic, which may limit generalizability today; that said, Head Start operations have largely returned to pre-COVID-19 conditions and ECE-based interventions continue to grow. Second, these data were collected in Northeastern US; other contexts may present distinct facilitators and barriers, based on state-level policies [66]. That said, Head Start is a federal program, which offers some generalizability and scalability of findings from the current study. Third, recall and social desirability bias may have been introduced, given that self-report surveys were administered in the context of the workplace; to limit this, surveys remained anonymous, and sample sociodemographic characteristics were not collected for those who completed the interviews, to help protect participant identities. Finally, it is possible that those who completed the interviews or surveys were systematically different from the larger Head Start staff population (thereby introducing selection bias); however, this is unlikely given that we collected data from a range of participants, exemplified by the range of years at Head Start and roles represented, for example.

This study also offers notable strengths. First, this study leverages multi-year data from a large sample of staff from *n* = 16 Head Start programs across two agencies, offering a larger sample size than most comparable studies. Second, this study reports on an important context for health interventions [67], with Head Start being the largest federally funded early childhood education program in the US, serving nearly one million children every year. Third, findings were generated at the intersection of both qualitative and quantitative data from diverse stakeholders (Head Start leadership, staff, and facilitators), who are underrepresented in the literature. This multimethod approach elucidated a richer understanding of their experiences implementing the intervention than would be possible through one data source alone.

## 5. Conclusions

In conclusion, our findings suggest that CBPR obesity prevention intervention facilitators in the context of ECEs include mission alignment, partner engagement in design, allocation of intervention-specific resources, and expressed leadership support. Barriers included strains imposed on staff workflow, a lack of shared responsibility, and challenges in coordinating CHL activities amidst competing Head Start programs. In response, interventionist designers should align activities with current ECE workflows, define mission alignment while distinguishing the unique value added by intervention activities, and allocate intervention-specific resources to ensure program continuity long-term.

## Figures and Tables

**Table 1:** Demographic characteristics of survey respondents (*n* = 144 Head Start staff and parents, Massachusetts, 2017–2019).

	Staff Readiness Survey (*n* = 119)	Facilitator Readiness Survey (*n* = 22)
	*n* (%)	*n* (%)
**Gender**		
Female	73 (61.3%)	22 (100%)
Male	44 (37.0%)	0 (0.0%)
**Age (years)**		
Mean (SD)	43.4 (11.9)	30.6 (9.56)
**Race and ethnicity**		
Hispanic	40 (33.6%)	6 (27.3%)
NH American Indian/Alaska Native	1 (0.8%)	0 (0.0%)
NH Asian	13 (10.9%)	2 (9.1%)
NH Black or African American	21 (17.6%)	7 (31.8%)
NH White or Caucasian	23 (19.3%)	5 (22.7%)
NH more than one race	1 (0.8%)	2 (9.1%)
Prefer not to answer	9 (7.6%)	0 (0.0%)
**Educational attainment**		
Some high school	0 (0.0%)	3 (13.6%)
High School/GED ^a^	0 (0.0%)	3 (13.6%)
Some college or technical school	14 (11.8%)	2 (9.1%)
Associate’s degree	41 (34.5%)	5 (22.7%)
Bachelor’s degree	41 (34.5%)	9 (40.9%)
Graduate degree	18 (15.1%)	0 (0.0%)
**Years at Head Start**		
<1 year	15 (12.6%)	1 (4.5%)
1–3 years	20 (16.8%)	15 (68.1%)
4–10 years	17 (14.3%)	0 (0.0%)
10–15 years	24 (20.2%)	1 (4.5%)
16+ years	36 (30.3%)	2 (9.1%)
**Role**		
Education or Disabilities staff	58 (48.7%)	N/A ^b^
Family Engagement, Nutrition, or Health staff	38 (31.9%)	N/A ^b^
Administrative staff	7 (5.9%)	N/A ^b^
Parent PConnect facilitator	N/A ^b^	7 (31.8%)
Staff PConnect facilitator	N/A ^b^	15 (68.2%)
Prefer not to answer	1 (0.8%)	0 (0.0%)

Responses may not add to 100% due to missingness. NH = non-Hispanic. Characteristics of those who participated in the staff training evaluations, interviews, and focus groups were not collected to protect staff anonymity. ^a^ GED = General Educational Development (GED). ^b^ N/A = not applicable, as response option(s) were not provided in the survey.

**Table 2:** Joint display used to compare and integrate findings from of qualitative interviews and surveys.

Themes	Interview Quotes	Survey Responses
Facilitators		
**[Theme 1: Mission alignment] Activities align** with Head Start mission	“I think, with [Head Start’s] mission [to support] families, I think [CHL] gives you one more way to connect with families—Leadership “[CHL] helps a lot because it help us to do a better connection with the parents and also improve my skills to work with some of the other facilitators, and also manage the time”—Staff “If you don’t take care of the basic needs of children and families, guess what? Doesn’t matter what you teach them—not gonna sustain. We’ve lost sight of that.”—Leadership “Honestly, we work with a population that some of these children do not eat. Do not eat. These parents are under enormous amount of stress even to get them to school, never mind try to find something healthy for them to eat. If we don’t…focus on [child health] forget about school readiness.”—Leadership “If you are a Head Start, true and true, [CHL] should be a priority.”—Leadership	40.3% strongly agreed and 52.9% agreed CHL addresses an important need for the community 95.5% of PConnect facilitators agreed that PConnect would help them connect with parents 68.2% of PConnect facilitators agreed it would help them learn more about health topics 95.5% of PConnect facilitators agreed it would help them gain skills with active listening 77.3% of PConnect facilitators agreed it would help them professionally
**[Theme 2: Engaging]** Participation in the design process results in high quality materials	“I feel like the resource map was a really great… tool that I could share with those specific staff members. It helped them support their families.”—Staff “The good thing is that whenever we would contribute ideas, suggestions, they were implemented… That’s been great. You guys have been very receptive in listening to everything the community has to say.”—Staff “Any changes that we suggested were done. In the meeting we all talked about it. Then the next meeting, we will see what they work on and results. We were agreeing on that’s what we want.”—Staff “I just have appreciated being involved in the project planning end of it and sort of seeing how parents are reacting and being involved in the CAB and the PConnect. I like having an environment where the parents are involved in that way. It feels more positive than a bunch of the staff sitting together and planning a project for parents. I think having the parents involved in the actual planning process is much more sustainable and applicable.”—Staff	63.3% strongly agreed and 34.3% agreed they would use the Healthy Habits brochures 81.3% agreed the Healthy Habits brochures are useful 33.3% use the Staff Talking points consistently
**[Theme 3: Available Resources]** Allocation of CHL-specific resources is necessary for success.	“I think that the support that [CHL coordinator] gives in terms of facilitating what goes on in the program is extremely helpful. She’s there. She’s around. She kind of takes the burden off people, so I think having that staff person that’s connected with Head Start and the project is really a good benefit.”—Leadership “If the grant is not there for us to have as much staff involved in the recruitment and talking to parents and staff about the program, it could be harder to get parents involved.”—Staff “We are running by a shoestring budget… you definitely need some funding to provide parents with the food and everything.”—Staff “[I] really do wish that we had more time and resources to kind of get parents more engaged. I think if there was one thing that I would suggest is maybe more involvement from the CHL staff to be able to support us in talking to parents about CHL.”—Staff	28.6% strongly agreed and 54.6% agreed they would be supported throughout CHL implementation 22.7% strongly agreed and 54.6% agreed they could handle challenges that might arise when implementing CHL
**[Theme 4: Relative priority]** Demonstrated support from leadership is necessary for staff buy-in and prioritization.	“If you don’t have the support from the director, it doesn’t work.”—Staff “I think, as leadership, too, in the central office, that it has to be seen as a priority in terms of school readiness and the work that we do.”—Leadership “It’s good to have the people who are helping with recruitment and who are frontline staff involved in CHL, but it’s also good to have people who are upper management to kind of understand what CHL is about because if the program director has an understanding of what’s happening.”—Staff “I think it needs to be a lot higher up. They need a lot more training or something, and that it is an important program ‘cause people brush it off a lot.”—Staff	28.6% strongly agreed and 54.6% agreed they are confident staff will be supported in CHL implementation 19.3% strongly agreed and 50.4% agreed that CHL is a priority at their program 54.5% of facilitators observed PConnect to be a high priority at the Head Start program
**Barriers**		
**[Theme 5: Deliverer opportunity]** Strain imposed on workflow.	“My supervisor might say that [CHL] has, potentially, taken away from other things… because it wasn’t technically my job description.”– Staff “I think it’s hard because we’re expecting the--it’s pretty much the staff who is implementing CHL also has to run this 10-week program and it’s too much time. Because they’re devoting 10 weeks of their time running this program, they’re not spending time doing other outreach to parents. I think there’s an imbalance of the time, so we’re not catching enough parents.”—Staff “It took more of my time because it was a new program that I got covered in and I had to do all my duties on my job, it takes more time, plus we have a staff meeting that we have to assist. It’s a lot.”—Staff “I feel like [CHL] needs to be something that is added in to our responsibilities, I guess. I don’t know if that makes sense.”—Staff	22.7% strongly agreed and 58.8% agreed that CHL fits within their current job tasks and responsibilities 4.2% strongly agreed and 31.1% agreed reported being constantly under pressure 7.6% strongly agreed and 40.3% agreed their coworkers show stress
**[Theme 6: Teaming]** Lack of shared responsibility amidst frequent turnover.	“We’ve lost a few staff over the last couple of years, and so being able to have multiple staff members who are trained, I think is gonna be key. Having a new group trained every year, if you don’t have someone who has seen it through who can be a mentor to other people, it can be tough.”—Staff “Incorporating staff, I think, needs to be on the front burner.”—Leadership “I think it needs to be more cohesive with staff being included. I’ve always learned that if staff were engaged, then I saw more happening.”—Leadership	64.5% strongly agreed & 34.3% agreed they understood their role in CHL 30.3% strongly agreed and 62.2% agreed they were committed to implementing CHL
**[Theme 7: Compatibility]** Challenges coordinating competing programs.	“I feel like…trying to schedule [CHL] around other programs, like the Parenting Journey-type thing that they offer, I feel like that’s the only, real problems with the CHL grant.”—Staff “We have classes, consults, parent meetings, different workshops so the parents want to do everything, but they can’t… that could be affecting attendance.”—Staff “They would be confused because we have so many meetings… Sometimes they were confused with having meetings, problems with canceled meetings, the [Head Start] meetings, the Head Start health advisory meetings.”—Staff “There were a lot of difficulties just with the staff at my school scheduling the room and a lot of things that made it very stressful for me to do.”—Staff	21.0% strongly agreed and 63.9% agreed they could help coordinate efforts 32.8% strongly agreed and 59.7% agreed they want to implement CHL

## Data Availability

The data presented in this study are available from the corresponding author upon request due to ethical restrictions.

## Supplementary Materials

The following supporting information can be downloaded at: https://www.mdpi.com/article/10.3390/nu17061063/s1, Table S1. Head Start staff and parent facilitator survey responses on implementation readiness and training experiences (Massachusetts, 2017–2019); Table S2. Head Start staff survey responses on CHL material utilization (*n* = 75, Massachusetts, 2017–2019); Table S3. Facilitator Training Survey (*n* = 22 Head Start staff and parent facilitators).

## Abbreviations

The following abbreviations are used in this manuscript:

CAB	Community Advisory Board
CBPR	Community-Based Participatory Research
CFIR	Consolidated Framework for Implementation Research
CHL	Communities for Healthy Living
ECE	Early care and education
