# Supplementary material for: “It Should Be a Priority”: Lessons Learned by Head Start Leaders, Staff, and Parent Facilitators Delivering a Multi-Site Parent-Centered Child Obesity Prevention Intervention
Source: Nutrients. 2025 Mar 18;17(6):1063. doi: 10.3390/nu17061063 (PMC11944454; doi:10.3390/nu17061063)
Supplement: Supplementary file 1 [file nutrients-17-01063-s001.zip › nutrients-3504919-supplementary.pdf]

Supplementary Materials

Table S1. Head Start staff and parent facilitator survey responses on implementation readiness and training experiences (Massachusetts, 2017-2019).

	Strongly Agree	Agree	Neutral	Disagree	Strongly Disagree
	**n (%)**	**n (%)**	**n (%)**	**n (%)**	**n (%)**
**Staff Readiness Survey (n=119 Head Start staff)**					
**Appropriateness**					
CHL addresses an important need in our community.	48 (40.3%)	63 (52.9%)	3 (2.5%)	0 (0.0%)	2 (1.7%)
CHL fits within my current job tasks and responsibilities.	27 (22.7%)	70 (58.8%)	14 (11.8%)	5 (4.2%)	1 (0.8%)
CHL will be effective in improving the health of our children and families.	55 (46.2%)	58 (48.7%)	3 (2.5%)	1 (0.8%)	1 (0.8%)
**Organizational climate**					
I am confident that I will be supported as I implement CHL.	34 (28.6%)	65 (54.6%)	15 (12.6%)	3 (2.5%)	1 (0.8%)
CHL is a priority for my program or program.	23 (19.3%)	60 (50.4%)	17 (14.3%)	10 (8.4%)	5 (4.2%)
No matter how much I do at work, there is always more to do.*	43 (36.1%)	56 (47.1%)	11 (9.2%)	7 (5.9%)	1 (0.8%)
Once I start a task at work, I am not given enough time to complete it.*	11 (9.2%)	41 (34.5%)	30 (25.2%)	30 (25.2%)	4 (3.4%)
I am constantly under pressure at work.*	5 (4.2%)	37 (31.1%)	32 (26.9%)	36 (30.3%)	7 (5.9%)
My coworkers often show signs of stress.*	9 (7.6%)	48 (40.3%)	25 (21.0%)	29 (24.4%)	5 (4.2%)
**Emotional engagement**					
I feel positive about my job.	46 (38.7%)	65 (54.6%)	6 (5.0%)	0 (0.0%)	2 (1.7%)
I am proud of my job.	59 (49.6%)	52 (43.7%)	6 (5.0%)	0 (0.0%)	2 (1.7%)
I am excited about my job.	50 (42.0%)	58 (48.7%)	7 (5.9%)	1 (0.8%)	2 (1.7%)
**Commitment**					
I am committed to implementing CHL.	36 (30.3%)	74 (62.2%)	6 (5.0%)	0 (0.0%)	2 (1.7%)
I want to implement CHL.	39 (32.8%)	71 (59.7%)	6 (5.0%)	0 (0.0%)	2 (1.7%)
I can help coordinate the different parts of CHL so implementation goes smoothly.	25 (21.0%)	76 (63.9%)	13 (10.9%)	2 (1.7%)	1 (0.8%)
I can handle the challenges that might arise when implementing CHL.	27 (22.7%)	65 (54.6%)	23 (19.3%)	2 (1.7%)	1 (0.8%)
**Staff Training Survey (n=166 Head Start staff)**					
**Clarity of role:** I understand my role in the CHL.	107 (64.5%)	57 (34.3%)	N/A	0 (0.0%)	2 (1.2%)
**Self-efficacy:** I feel confident that I can use the Staff Talking Points.	91 (54.8%)	72 (43.4%)	N/A	1 (0.6%)	1 (0.6%)
**Intention:** I plan on helping recruit parents for the PConnect program (e.g. by telling parents about it, by handing out flyers, etc.).	96 (57.8%)	67 (40.4%)	N/A	2 (1.2%)	1 (0.6%)

**Intention:** I will use the Healthy Habits brochures as a resource for myself or if parents are looking for more information.	105 (63.3%)	57 (34.3%)	N/A	2 (1.2%)	1 (0.6%)
**Facilitator Training Survey (n=22 Head Start staff and parent facilitators)**					
**Trainer evaluation**					
The trainer(s) created a comfortable learning environment	22 (100%)	0 (0.0%)	0 (0.0%)	0 (0.0%)	0 (0.0%)
The trainer(s) were well prepared	21 (95.5%)	1 (4.5%)	0 (0.0%)	0 (0.0%)	0 (0.0%)
The trainer(s) gave clear explanations	20 (90.9%)	2 (9.1%)	0 (0.0%)	0 (0.0%)	0 (0.0%)
The trainer(s) modeled how to be a good facilitator	22 (100%)	0 (0.0%)	0 (0.0%)	0 (0.0%)	0 (0.0%)
**Self-efficacy**					
I am prepared to lead a group of parents	16 (72.7%)	5 (22.7%)	0 (0.0%)	0 (0.0%)	0 (0.0%)
I am prepared to run the activities we covered during the training	15 (68.2%)	6 (27.3%)	0 (0.0%)	0 (0.0%)	0 (0.0%)
I am prepared to work with my co-facilitator.	17 (77.3%)	4 (18.2%)	0 (0.0%)	0 (0.0%)	0 (0.0%)
I already know what I need to know about cultures of parents who will be in my PConnect group.	12 (54.5%)	10 (45.5%)	0 (0.0%)	0 (0.0%)	0 (0.0%)
I am ready to learn from parents whose culture is different from my own	20 (90.9%)	2 (9.1%)	0 (0.0%)	0 (0.0%)	0 (0.0%)
Parents from cultures different from my own have a lot to offer the program.	19 (86.4%)	3 (13.6%)	0 (0.0%)	0 (0.0%)	0 (0.0%)
I have specific skills for working with individuals from cultures different from my own.	16 (72.7%)	2 (9.1%)	0 (0.0%)	3 (13.6%)	0 (0.0%)
**Appropriateness**					
PConnect addresses an important need in our community.	17 (77.3%)	5 (22.7%)	0 (0.0%)	0 (0.0%)	0 (0.0%)

Table S2. Head Start staff survey responses on CHL material utilization (n=75, Massachusetts, 2017-2019).

	Yes	Sometimes	No	N/A
	**n (%)**	**n (%)**	**n (%)**	**n (%)**
**Tool 1: Staff Talking Points**				
The Staff Talking Points are a useful tool.	36 (48.0%)	20 (26.7%)	1 (1.3%)	14 (18.7%)
I use the Staff Talking Points.	25 (33.3%)	24 (32.0%)	10 (13.3%)	12 (16.0%)
**Tool 2: Healthy Habits brochure**				
The Healthy Habits brochures are useful.	61 (81.3%)	9 (12.0%)	1 (1.3%)	2 (2.7%)
I use the Healthy Habits brochures with parents.	50 (66.7%)	15 (20.0%)	4 (5.3%)	6 (8.0%)
**Tool 3: Online Neighborhood Resource Map**				
I have used the online Neighborhood Resource Map.	31 (41.3%)	19 (25.3%)	20 (26.7%)	5 (6.7%)
I showed the Neighborhood Resource Map to parents.	38 (50.7%)	17 (22.7%)	12 (16.0%)	7 (9.3%)
**Tool 4: Posters**				
The CHL posters at my program are useful.	50 (66.7%)	12 (16.0%)	4 (5.3%)	7 (9.3%)
Parents look at the posters and flyers hanging up at my program.	36 (48.0%)	27 (36.0%)	3 (4.0%)	8 (10.7%)
**Tool 5: Checklist**				
I use the checklist for completing the Nutrition and Physical Activity Questionnaire.	26 (34.7%)	13 (17.3%)	17 (22.7%)	17 (22.7%)
I use the checklist for meeting with families who have children in the >85th percentile.	25 (33.3%)	10 (13.3%)	18 (24.0%)	20 (26.7%)

Table S3. Facilitator Training Survey (n=22 Head Start staff and parent facilitators).

	**n (%)**
**Past experience facilitating a class, group, or workshop**	
Yes	16 (72.7%)
No	6 (27.3%)
**Anticipated benefits of facilitating PConnect**	
It will help me connect with parents.	21 (95.5%)
It will help me learn more about topics related to health.	15 (68.2%)
I will gain skills in group facilitation such as active listening, organization, and communication.	21 (95.5%)
It will help me professionally.	17 (77.3%)
**Perceived prioritization of PConnect at the Head Start program**	
High	12 (54.5%)
Medium	10 (45.5%)
Low	0 (0.0%)
**Perceived PConnect fit current work tasks or job description**	
Yes, it fits	16 (72.7%)
Somewhat fits	4 (18.2%)
No, it does not fit	2 (9.1%)
**Likelihood of facilitating another PConnect program in the future**	
Very likely	14 (63.6%)
Likely	8 (36.4%)
**Anticipated effectiveness of PConnect in improving participant health**	
Very effective	13 (59.1%)
Somewhat effective	6 (27.3%)
Not effective	2 (9.1%)
